# Validation of cellular tests for Lyme borreliosis (VICTORY) study

**DOI:** 10.1186/s12879-019-4323-6

**Published:** 2019-08-20

**Authors:** F. R. van de Schoor, M. E. Baarsma, S. A. Gauw, L. A. B. Joosten, B. J. Kullberg, C. C. van den Wijngaard, J. W. Hovius

**Affiliations:** 1 Radboudumc, Department of Internal Medicine, Radboud Center for Infectious Diseases (RCI) and Radboud Institute of Health Sciences (RIHS), P.O. Box 9101, 6500 HB Nijmegen, The Netherlands; 2 Amsterdam UMC, University of Amsterdam, Center for Experimental and Molecular Medicine, Amsterdam Institute of Infection and Immunology, P.O. Box 22660, 1100 DD Amsterdam, The Netherlands; 3 National Institute for Public Health and the Environment (RIVM), Center of Infectious Disease Control, P.O. Box 1, 3720 BA Bilthoven, The Netherlands

**Keywords:** Lyme disease, Borreliosis, Erythema Migrans, Borrelia, Validation, Cellular tests, Serology, Study protocol

## Abstract

**Background:**

Lyme borreliosis (LB) is a tick-borne disease caused by spirochetes belonging to the *Borrelia burgdorferi* sensu lato species. Due to a variety of clinical manifestations, diagnosing LB can be challenging, and laboratory work-up is usually required in case of disseminated LB. However, the current standard of diagnostics is serology, which comes with several shortcomings. Antibody formation may be absent in the early phase of the disease, and once IgG-seroconversion has occurred, it can be difficult to distinguish between a past (cured or self-cleared) LB and an active infection. It has been postulated that novel cellular tests for LB may have both higher sensitivity earlier in the course of the disease, and may be able to discriminate between a past and active infection.

**Methods:**

VICTORY is a prospective two-gate case-control study. We strive to include 150 patients who meet the European case definitions for either localized or disseminated LB. In addition, we aim to include 225 healthy controls without current LB and 60 controls with potentially cross-reactive conditions. We will perform four different cellular tests in all of these participants, which will allow us to determine sensitivity and specificity. In LB patients, we will repeat cellular tests at 6 weeks and 12 weeks after start of antibiotic treatment to assess the usefulness as ‘test-of-cure’. Furthermore, we will investigate the performance of the different cellular tests in a cohort of patients with persistent symptoms attributed to LB.

**Discussion:**

This article describes the background and design of the VICTORY study protocol. The findings of our study will help to better appreciate the utility of cellular tests in the diagnosis of Lyme borreliosis.

**Trial registration:**

NL7732 (Netherlands Trial Register, trialregister.nl).

**Electronic supplementary material:**

The online version of this article (10.1186/s12879-019-4323-6) contains supplementary material, which is available to authorized users.

## Background

Lyme borreliosis (LB) is a widely prevalent infectious disease in the Northern Hemisphere with potentially major health consequences [1, 2]. The causative agent of LB is one of several species of bacteria from the *Borrelia burgdorferi* sensu lato complex, which require the tick as a vector for transmission. LB can be arbitrarily divided into early localized, early disseminated and late (disseminated) manifestations [3]. Common manifestations in Europe include erythema migrans (EM), Lyme neuroborreliosis (LNB), Lyme arthritis and acrodermatitis chronica atrophicans (ACA) [4, 5].

The diagnosis of LB is primarily based on clinical features, usually combined with serological testing or, when indicated, direct detection of the pathogen in specific bodily materials. As holds true for many serological tests for infectious diseases, *Borrelia* serology comes with several shortcomings. Sensitivity and specificity depend on several factors, which include the disease duration and type of manifestation. Moreover, an inherent issue with serological testing is the fact that antibody formation takes time and can be aborted after antibiotic treatment [6, 7]. A recent review by Leeflang and colleagues found the overall sensitivity of ELISAs and immunoblots used in Europe to be up to 95% for late manifestations, but as low as 50% for early localized disease (EM) [8]. Specificity ranged from 80 to 95% [8]. An additional problem arises once IgG-seroconversion has occurred, since antibodies oftentimes remain present in the blood for many years, even after the infection has long been cured or self-cleared [7, 9, 10]. These test characteristics result in diagnostic dilemmas in several clinical situations for both patients and physicians.

Some LB patients report persistent symptoms, with the exact percentage varying per manifestation and population assessed [11–14]. These complaints cause a substantial disease burden in the Netherlands [15]. These symptoms may develop after early, disseminated or late manifestations of LB, despite recommended antibiotic treatment. These symptoms, which mainly include fatigue, general malaise, musculoskeletal pain and neurological symptoms, are more likely to occur when there has been a delay in proper antibiotic treatment [16, 17]. For this reason, an early diagnosis of LB has clear treatment benefits. Antibiotic treatment failure, defined as microbiologically confirmed persisting *B. burgdorferi* s.l. infection, is uncommon, but has been described [18]. Therefore, a discussion may ensue about whether complaints are caused by a persisting infection (requiring antibiotic treatment), if these symptoms are post-infectious or not even related to LB. Serology cannot make this distinction once seroconversion has occurred.

Cellular tests for LB have been proposed as a (partial) solution to some of these dilemmas. These tests utilize the cellular part of the immune system and have been in use for the diagnosis of tuberculosis for several decades [19]. These in vitro assays function by stimulating patient leukocytes with pathogen-specific antigens and measuring the resulting cellular response. Previous studies have examined the diagnostic value of various types of cellular tests for LB and have yielded varying results [20–32]. A more recent study by Callister and colleagues found that the sensitivity of a cellular assay for LB based on interferon-gamma (IFN-γ) was higher than that of serology in patients with early localized LB (an EM). In addition, the authors showed that the IFN-γ response waned within months after antibiotic treatment [33]. This supports the hypothesis that cellular tests may have a higher sensitivity than serology earlier in the course of disease, and that they may be used a test-of-cure (i.e. positive before treatment and negative after successful treatment). For patients who have long-lasting –sometimes invalidating– complaints that are attributed to LB, a test that better distinguishes between a persistent *Borrelia-*infection, post-treatment Lyme borreliosis syndrome (PTLBS) or an altogether different condition could be of great benefit, and could thus guide patient management.

The aforementioned studies investigating cellular tests for LB had important shortcomings. They lacked clear case definitions, were performed in only a small number of participants, or they lacked comparisons with adequate control groups. In addition, many of these studies were performed by the developers of the tests, with insufficient safeguards to prevent a potential conflict of interest. Conversely, the present VICTORY study has been designed to assess the diagnostic performance of various cellular tests for LB in well-defined groups of patients with acute LB and controls by means of a two-gate case-control design. Additionally, we will study the utility of these tests in an observational cohort of patients with prolonged symptoms attributed to LB. In this paper, we describe the study protocol in detail.

## Methods/design

### Study design

This is a multi-center prospective two-gate case-control study with 1 year of follow-up to assess the diagnostic parameters of cellular tests for LB. In addition, an observational prospective cohort is included to investigate the performance of cellular tests in patients with persistent symptoms attributed to LB. This study entails a collaboration between the Dutch National Institute for Public Health and the Environment (RIVM, Bilthoven, the Netherlands) and the clinical expert centers for Lyme borreliosis at the Amsterdam UMC (formerly AMC, Amsterdam, the Netherlands), and the Radboud university medical center (Radboudumc, Nijmegen, the Netherlands). The study has been approved by the medical ethics committee (MEC) of the Amsterdam UMC (registered under no. NL63961.018.18), and is conducted according to the principles of the Declaration of Helsinki.

### Study population

We will enroll three groups in the case-control study. Cases are 150 adults from the Netherlands with acute confirmed LB, included before or just after start of antibiotic treatment (Table 1). Our study uses well-established case definitions that largely match the European case definitions as described by Stanek and colleagues [3]. LB patients have early localized, early disseminated, or late disseminated LB. We are also recruiting 225 participants from the general population, who have no current complaints of LB. These participants function as healthy controls in the validation study. Healthy controls are matched with cases by age, gender, and – in order to correct for tick bites – by geographical region. We are enrolling a separate control cohort of 60 participants with medical conditions that are potentially cross-reactive with LB in these cellular tests. These medical conditions include other spirochetal infections (leptospirosis, syphilis), infection with Epstein-Barr virus (EBV) or cytomegalovirus (CMV), and rheumatoid arthritis (RA). Furthermore, the cellular tests are performed in an observational cohort of participants with persistent symptoms attributed to LB. All adult participants visiting the Lyme outpatient clinics of the Amsterdam UMC or Radboudumc are eligible for the observational cohort and participants are included consecutively. Recruitment has started in May 2018.

**Table 1:** Inclusion and exclusion criteria

**Patients with confirmed Lyme borreliosis**	
Inclusion criteria:	
Patient ≥18 years with confirmed proven or probable, early localized or disseminated Lyme borreliosis manifestation.^a^	
In case of an EM reported at www.tekenradar.nl, the EM has been present < 3 months and the clinical diagnosis has been confirmed by the general practitioner.	
Subjects live or stay on the mainland of the Netherlands.	
Exclusion criteria:	
Subjects unable to provide informed consent or do not have sufficient proficiency in the Dutch language.	
Subjects having started antibiotic treatment > 4 days before inclusion (for subjects included online) or > 7 days before inclusion (for subjects included through the clinical expert centers).	
Subjects having ongoing signs or symptoms attributed to a previous episode of Lyme borreliosis.	
**Healthy controls**	
Inclusion criterium:	
Participant ≥18 years old.	
Exclusion criteria:	
Uncontrolled HIV-infection, if known. This is defined as an HIV-1 viral load > 40 copies/ml and/or CD4+ count < 500 × 10^6^ cells/liter in the past 12 months.	
Active syphilis or leptospirosis, an active infection with EBV/CMV, or an auto-immune disease, if known.	
Current LB with typical symptoms. A past episode or *Borrelia* seropositivity is not an exclusion criterium.	
Immunomodulating medication including > 7.5 mg prednisone daily, methotrexate, biologicals. Medication such as hydroxychloroquine, sulfazalazine or NSAIDs are accepted.	
Known immunodeficiency, hematologic malignancies in the medical history or chemotherapy during the past year.	
Subjects unable to provide informed consent or do not have sufficient proficiency in the Dutch language.	
**Potentially cross-reactive controls**	
Inclusion criteria:	
Participant ≥18 years old.	
AND	
1. Patients with syphilis:	
EITHER clinical symptoms suspected of secondary syphilis, in combination with a positive RPR card POCT result.	
OR early latent syphilis infection with VDRL/RPR ≥ 1:32.	
2. Patients with Epstein Barr-virus or cytomegalovirus:	
Compatible signs and symptoms.	
Positive IgM or Paul-Bunnell of EBV/CMV on plasma.	
3. Patients with leptospirosis:	
Compatible signs and symptoms.	
Positive serology (MAT), preferably confirmed by culture or PCR.	
4. Patients with autoimmune diseases:	
EITHER confirmed rheumatoid arthritis.	
OR other autoimmune disorders diagnosed according to the leading guidelines.	
Exclusion criteria:	
Uncontrolled HIV-infection, if known. This is defined as an HIV-1 viral load > 40 copies/ml and/or CD4+ count < 500 × 10^6^ cells/liter in the past 12 months.	
More than one of the listed potentially cross-reactive conditions, if known.	
Ever an episode of LB, ever treated for LB or a known tick bite in the past 6 months.	
Immunomodulating medication including > 7.5 mg prednisone daily, methotrexate, biologicals. Medication such as hydroxychloroquine, sulfazalazine or NSAIDs are accepted.	
Known immunodeficiency, hematologic malignancies in the medical history or chemotherapy during the past year.	
Subjects unable to provide informed consent or do not have sufficient proficiency in the Dutch language.	
**Observational cohort**	
Inclusion criteria:	
Participants ≥18 years old.	
Presenting at the specialized Lyme centers of the Amsterdam UMC or Radboudumc.	
Exclusion criterium:	
Subjects unable to give informed consent or do not have sufficient proficiency in the Dutch language.	

^a^ Specific criteria for each manifestation of confirmed Lyme borreliosis are identical to the criteria listed in the LymeProspect study protocol [34].

### Recruitment, inclusion and follow-up of participants

The majority of LB cases are recruited as part of the LymeProspect study, which is also a collaborative effort of the RIVM, Amsterdam UMC and Radboudumc (Netherlands Trial Register: NTR4998/NL4744) [34]. Cases with disseminated LB are additionally recruited under the current protocol. The recruitment procedures, and in- and exclusion criteria for cases are identical in both protocols.

**Fig. 1:**
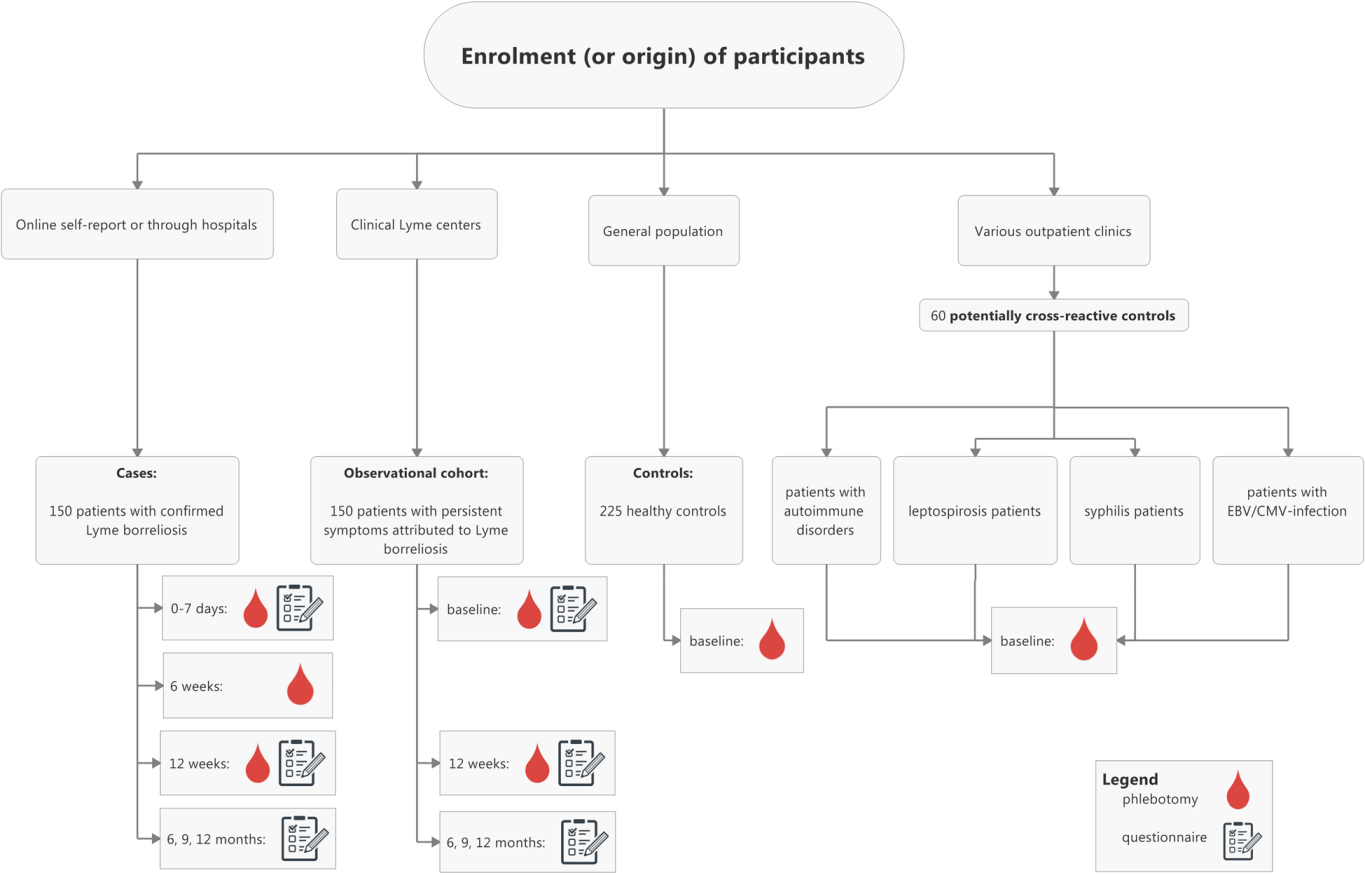
Flowchart of study design

Recruitment, inclusion and follow-up of LB cases occur both online and at the LB clinical expert centers (Fig. 1). Most LB cases are recruited through the website Tekenradar.nl. This national and secured online enrollment platform is operated by the RIVM. Patients can visit the website on their own initiative, or after having been referred to it by their general practitioner (GP). Patients thus recruited primarily have an EM as manifestation of LB, but other forms of confirmed LB may be included through Tekenradar.nl as well. Written informed consent is obtained from eligible participants. Importantly, the participant’s medical doctor is consulted to verify the diagnosis after online enrollment. Blood collection can be done locally or in the participating hospitals and is performed at baseline, after 6 weeks and facultatively after 12 weeks. A secondary inclusion route for cases is through the LB clinical expert centers, after evaluation by one of the investigators (Table 2).

**Table 2:** Data collection and measurements for Lyme borreliosis cases

	Baseline	10 days	2 weeks	6 weeks	3 months	6 months	9 months	12 months
Written information and informed consent	X							
Baseline characteristics	X							
Physical examination	X^a^				X^ac^	X^ac^	X^ac^	X^ac^
Recording LB manifestation, treatment and concomitant medication	X		X^b^		X	X	X	X
Recording adverse events	X		X^b^		X	X		X^a^
Laboratory measurements								
*B. burgdorferi* s.l. serology	X	X^ac^		X	X^ac^			
Spirofind Revised	X			X	X^c^			
QuantiFERON-LB	X			X	X^c^			
iSpot Lyme	X			X	X^c^			
LTT-MELISA	X			X	X^c^			
Biopsies from skin manifestations (optional)	X^cd^			X^cd^	X^cd^			
Questionnaires								
CIS1 (subscale fatigue), SF-36 (subscale pain), CFQ	X				X^e^	X	X^e^	X
Clinical parameters: PHQ-15, SF-36 (subscale physical functioning and subscale social functioning), TiC-P (health care use and absenteeism of work)	X				X	X	X	X
Cognitive-behavioral parameters: brief IPQ, CBRSQ, HADS, SES-F, PCS, IPAQ	X				X	X		
Comorbidities: TiC-P (co-morbidity list)	X							X
Comorbidities: PREDIS	X							

For explanation of abbreviations, see the main text

^a^Patients included through the clinical expert centers for Lyme borreliosis only

^b^Patients included through the website www.tekenradar.nl only. ^c^These visits and laboratory measurements can be left out if patients are not able or not willing to. This is regarded as an allowed deviation from the protocol

^d^Three skin biopsy samples of the skin lesion at baseline and if still present at 6 weeks or at 3 months time point. ^e^CIS questionnaire only short version, to limit the burden for patients

Controls are only recruited under the current protocol. Healthy controls are recruited in two ways. Firstly, from the general population, i.e. from a cohort that has previously been recruited to fill out questionnaires as a population control for other LB-related studies. Additionally, we recruit healthy controls through local laboratories, GP’s offices and outpatient clinics. After obtaining written informed consent, blood is drawn only at baseline (Table 3). Next to inclusion through the Amsterdam UMC or Radboudumc, potentially cross-reactive controls are recruited through various medical institutions. Persons with syphilis may also be recruited through the sexually transmitted infections (STI) clinic of the Amsterdam Public Health Service. Persons with an autoimmune disease may be recruited from the Sint Maartenskliniek for rheumatology in Nijmegen. Persons with leptospirosis and an infection with EBV/CMV are only recruited from the Amsterdam UMC or Radboudumc. After obtaining written informed consent, blood is drawn only at baseline (Table 3).

**Table 3:** Data collection and measurements for healthy controls and potentially cross-reactive controls

	Baseline	3 months
Written information and informed consent	X	
Baseline characteristics	X	
Recording adverse events	X	X
Laboratory measurements		
*B. burgdorferi* s.l. serology	X	
Spirofind Revised	X	
QuantiFERON-LB	X	
iSpot Lyme	X	
LTT-MELISA	X	

For explanation of abbreviations, see the main text

Participants for the observational cohort with persistent symptoms attributed to LB are recruited from the LB clinical expert centers of the Amsterdam UMC or Radboudumc. After obtaining written informed consent, blood is drawn at baseline and after 12 weeks (Table 4).

**Table 4:** Data collection and measurements for the observational cohort

	Baseline	6 weeks	3 months	6 months	9 months	12 months
Written information and informed consent	X					
Baseline characteristics	X					
Physical examination	X					
Recording LB manifestation, treatment and concomitant medication	X		X	X	X	X
Recording adverse events	X		X	X		X
Laboratory measurements						
*B. burgdorferi* s.l. serology	X		X			
Spirofind Revised	X		X			
QuantiFERON-LB	X		X			
iSpot Lyme	X		X			
LTT-MELISA	X		X			
Questionnaires						
CIS1 (subscale fatigue), SF-36 (subscale pain), CFQ	X		X^a^	X	X^a^	X
Clinical parameters: PHQ-15, SF-36 (subscale physical functioning and subscale social functioning), TiC-P (health care use and absenteeism of work)	X		X	X	X	X
Cognitive-behavioral parameters: brief IPQ, CBRSQ, HADS, SES-F, PCS, IPAQ	X		X	X		
Comorbidities: TiC-P (co-morbidity list)	X					X
Comorbidities: PREDIS	X					

For explanation of abbreviations, see the main text

^a^CIS questionnaire only short version, to limit the burden for patients

### Epidemiological and clinical measurements

For LB cases and for participants in the observational cohort, standard demographical characteristics, comorbidities and details on previous tick exposure and previous or current episodes of LB are reported at baseline. During follow-up, new tick bites or new episodes of LB are recorded, as well as new non-LB-related medical diagnoses. Photographs of skin manifestations of LB are obtained for blinded evaluation by independent experts. Participants who are included online can upload these pictures themselves. Cases with confirmed LB who have a skin manifestation have the option of giving additional informed consent for skin biopsies to be taken (Table 2). For all controls, standard demographical characteristics, comorbidities and details on any previous episodes of LB are recorded only at baseline (Table 3).

### Laboratory measurements

Four cellular tests for LB will be performed: the Spirofind Revised (Oxford Immunotec), the QuantiFERON-LB (QIAGEN Sciences), the Lyme *i*Spot (Autoimmun Diagnostika / Genome Identication Diagnostics), and the Lymphocyte Transformation Test-Memory Lymphocyte Immunostimulation Assay (LTT-MELISA, InVitaLab). Details of the various cellular tests are provided in the Additional file 1. The *B. burgdorferi* s.l. C6 ELISA (Oxford Immunotec) is performed as serological test. All positive and indeterminate C6 ELISA results will be confirmed by IgM and IgG immunoblot (Mikrogen), according to guideline recommendations [35]. All samples are processed blinded, i.e. without any markings related to the identity of the participant, type of participant or time point.

### Questionnaires

Confirmed LB cases and participants in the observational cohort will also be asked to fill out online questionnaires through Tekenradar.nl. These will be filled out at inclusion, and after 3, 6, 9 and 12 months and cover a wide array of determinants of health, including assessment of symptoms, disabilities, cognitive-behavioral variables, co-morbid disorders and pre-existing symptoms. These questionnaires are validated and have Dutch norm scores, which enables us to use cutoff scores to determine the severity of symptoms. These questionnaires are specified in the Additional file 1.

### Outcome measures and data analysis

The primary outcome measures are the diagnostic parameters of the cellular tests in cases versus healthy controls. We will use the manufacturer-prescribed reference values for interpretation (positive, indiscriminate, negative), and perform a receiver operating characteristic (ROC-) analysis to assess potential new cutoffs. From this, we will calculate the diagnostic parameters (sensitivity and specificity) of the studied tests.

Secondary outcome measures are: i) the comparisons of the diagnostic parameters of the various cellular tests, ii) the correlation of cellular tests with serology results, and iii) the effectiveness of the cellular tests as a test-of-cure. The latter outcome is determined by correlating the cellular responses in LB participants over time to various determinants of health during the one-year follow-up period. Finally, we will assess the performance of the cellular tests and clinical outcomes in the observational cohort of patients with persistent symptoms attributed to LB.

### Sample size

We consider cellular tests to be an improvement over serology if they increase the sensitivity of laboratory testing for early LB by 20%, with specificity not dropping below 90%. Inclusion of 100 cases would result in a power of 97% (alpha < 0.05) to detect a significant improvement in sensitivity of 20%. Accounting for loss-to-follow-up, and the fact that the improvement in sensitivity may actually be smaller, we have chosen to include 150 cases in our case-control study. This would still yield a power of 85% (alpha < 0.05) to detect an improvement in sensitivity of 10–15%. We will additionally include 225 healthy controls and 60 potentially cross-reactive controls in the case-control study. For the observational cohort of patients with persistent signs and symptoms attributed to LB, we aim to include 150 participants. Details on the power calculations are provided in the Additional file 1.

## Discussion

The VICTORY study evaluates the diagnostic parameters of cellular tests for LB and additionally assesses their performance in an observational cohort of patients with persistent symptoms attributed to LB.

Cellular tests for LB may possibly help in clinical decision making for LB-related problems. National and international guidelines dictate that an EM is a clinical diagnosis and that serological tests should not be performed, as the chance of a false-negative result is considerable [3, 35]. However, the skin lesion is frequently atypical, and in approximately 50% of the cases there is no known history of a tick bite [36, 37], which makes the diagnosis considerably more difficult. Furthermore, the clinical spectrum of early LB can be broad, especially in the United States, where subfebrile temperature and other systemic symptoms are more common in early LB [36, 38, 39]. In such situations, a test with a higher sensitivity and specificity early in disease may lead to an earlier diagnosis.

Several studies have reported on the potential of cellular tests for LB. However, these studies have important shortcomings, which have also been discussed by others [40–43]. Nonetheless, several cellular tests for LB are commercially available in Europe. In the absence of a thorough assessment of the diagnostic parameters of these commercially available tests, clinicians cannot reliably use the test results in their clinical decision making process. The results of any such unvalidated tests do, however, give the patient an expectation of health or illness. This underscores the urgent need for a proper validation study for cellular tests for LB.

Our study is unprecedented compared to other studies on cellular tests for LB, as the patient populations and diagnoses are strictly defined according to consensus criteria, the study includes healthy controls from the general population and potentially cross-reactive controls, and is appropriately powered. Furthermore, a variety of cellular tests will be compared in parallel. Finally, this study has been designed and is currently being performed in direct collaboration with patient representatives taking into account the patients’ perspective.

As there is no universally applicable reference standard for LB, it is challenging to set up a sufficiently powered one-gate cohort study to assess the diagnostic parameters of the cellular tests under study [44]. However, in our study, the strict case definitions and physician confirmation of diagnosis function as a surrogate for this missing reference standard.

In conclusion, the VICTORY study is a hybrid study consisting of a prospective two-gate case-control study to assess the diagnostic parameters of multiple cellular tests for LB, and an observational prospective cohort study to assess their clinical application in an outpatient setting.

## Additional file

Additional file 1: (1) Specifics of laboratory measurements. (2) Questionnaires. (3) Sample size calculation. (PDF 378 kb)

## Data Availability

Not applicable.

## Abbreviations

ACA: Acrodermatitis chronica atrophicans
AMC: Academic Medical Center
Amsterdam UMC: Amsterdam University Medical Centers
CBRSQ: Cognitive Behavioural Responses to Symptoms Questionnaire
CD4: Cluster of differentiation 4
CFQ: Cognitive failure questionnaire
CIS: Checklist Individual Strength
CMV: Cytomegalovirus
DbpB: Decorin-binding protein B
EBV: Epstein-Barr virus
ELISA: Enzyme-Linked Immunosorbent Assay
ELISPOT: Enzyme-linked immunospot
EM: Erythema migrans
GP: General practitioner
HADS: Hospital Anxiety and Depression Scale
HIV: Human immunodeficiency virus
IFA: Immunofluorescence assay
IFN-γ: Interferon-gamma
IgG: Immunoglobulin G
IgM: Immunoglobulin M
IL: Interleukin
IPAQ: International Physical Activity Questionnaire
IPQ: Illness perception questionnaire
LB: Lyme borreliosis
LNB: Lyme neuroborreliosis
LTT-MELISA: Lymphocyte Transformation Test-Memory Lymphocyte Immunostimulation Assay
MAT: Microscopic agglutination test
MEC: Medical ethics committee
NSAID: Non-steroid anti-inflammatory drug
OspA: Outer-surface protein A
OspC: Outer-surface protein C
PBMC: Peripheral blood mononuclear cell
PCR: Polymerase chain reaction
PCS: Pain Catastrophizing Scale
PHQ: Patient Health Questionnaire
POCT: Point-of-care test
PTLBS: Post-treatment Lyme borreliosis syndrome
QFN-LB: Quantiferon-Lyme Borreliosis
RA: Rheumatoid arthritis
Radboudumc: Radboud university medical center
RIVM: Dutch National Institute for Public Health and the Environment
ROC: Receiver operating characteristic
RPR: Rapid plasma regain
s.l.: Sensu lato
SES: Self-Efficacy Scale
SF-36: SF-36 Health Status Inventory
SFUs: Spot-forming units
SI: Stimulation index
STI: Sexually transmitted infections
Tic-P: Trimbos/iMTA questionnaire for Costs associated with Psychiatric Illness
VDRL: Venereal disease research laboratory
