# Supplementary material for: Validation of cellular tests for Lyme borreliosis (VICTORY) study
Source: BMC Infect Dis. 2019 Aug 20;19:732. doi: 10.1186/s12879-019-4323-6 (PMC6700813; doi:10.1186/s12879-019-4323-6)
Supplement: Supplementary file 1 — (1) Specifics of laboratory measurements. (2) Questionnaires. (3) Sample size calculation. (PDF 378 kb) [file 12879_2019_4323_MOESM1_ESM.pdf]

Additional files

1. Laboratory measurements

Spirofind Revised

The *Spirofind Revised* (hereafter Spirofind) is a pre-market test that will be performed in part in-house at the AMC and Radboudumc, and in part on-site at the facilities of Oxford Immunotec in Oxford, the United Kingdom. It uses 6 ml of heparinized blood.

The test follows the analogy of Oxford Immunotec's patented *in vitro* diagnostic T-SPOT®.*TB*, as currently in use for the diagnosis of tuberculosis. It uses an enzyme-linked immunospot (ELISPOT) platform. The test is performed by isolating peripheral blood mononuclear cells (PBMCs) from a whole blood sample using specialized tubes containing Ficoll® (Leucosep). After isolation, the concentration of PBMCs is standardized using a Beckman Coulter Counter and cells are incubated with a mix of bacteria from several *Borrelia* spp. and recombinant *Borrelia* antigens, as well as with a negative and a positive control. Incubation is done in sterile 96-wells culture plates. These plates are pre-coated with antibodies to IFN-γ. After incubation, the plates are washed and developed as instructed by the manufacturer. The resulting spot-forming units (SFUs) are fixated and the plates stored, until they are sent blinded and batchwise to the facilities of Oxford Immunotec for further analysis. The results of the Spirofind are reported in SFUs, which are counted using a specialized automated counter and analysis software. Results are interpreted following the manufacturer's instructions.

Quantiferon-LB

The *Quantiferon-LB* (hereafter QFN-LB) is a test candidate under development, performed in-house at the AMC and Radboudumc. It uses 4 ml of heparinized blood.

The QFN-LB is performed essentially as previously described by Callister and colleagues [1]. It is based on QIAGEN's patented QuantiFERON technology, as currently in use for the diagnosis of tuberculosis using the QuantiFERON-TB Gold. The QFN-LB works by stimulating effector T cells of a patient *in vitro* with specific antigens and quantifying the resulting IFN-γ in plasma. The antigens used in the QFN-LB are proprietary recombinant proteins that are higly conserved (>80% identity) among the several *Borrelia* spp., while being distinct from non-*Borrelia* proteins (<50% identity) [1]. Whole blood is collected from a patient in a heparin tube and then transferred to specialized containers, containing antigen, pokeweed mitogen (positive control) or negative control. Samples are incubated in these containers at 37°C for 24 hours, after which the cells are spun down and the supernatant is collected. The supernatants are stored at -20°C and analysed batchwise by quantifying

the concentration of IFN-γ using an ELISA as instructed by the manufacturer. Results are interpreted following the manufacturer’s instruction.

iSpot

The two-colour iSpot is a test that is currently commercially available in Germany, having been developed by the company AID (also known as GenID). The iSpot is performed on-site at the facilities of AID/GenID in Strassberg, Germany. It uses 10 ml of heparinized blood.

The iSpot uses an ELISPOT technique. The technique used for the present study is identical to the techniques used in daily clinical practice at AID/GenID’s facilities. Whole blood samples are incubated with *Borrelia* lysate (B31) and a mix of recombinant *Borrelia* proteins (OspC, DbpB and OspA), as well as with a negative and a positive control. The production of two cytokines by T cells, namely IFN-γ and interleukin-2 (IL-2), is then assessed using the so-called ‘traffic light principle’. After incubation and development, wells that contain a high concentration of IFN-γ and a low concentration of IL-2 turn red, which according to the manufacturer indicates the presence of mainly effector T cells, which would be suggestive of an active infection. A reversed concentration of IFN-γ and IL-2 is indicated by a green colour, suggesting a higher concentration of memory T cells, indicating a past infection. A balanced concentration of IFN-γ and IL-2 is said to indicate a latent infection. Test results are interpreted following the manufacturer’s instruction.

LTT-MELISA

The Lymphocyte Transformation Test-Memory Lymphocyte Immunostimulation Assay (LTT-MELISA), is also currently commercially available in Germany. The LTT-MELISA is performed on-site at the facilities of Invitalab in Neuss, Germany. It uses 27 ml of blood in citrate.

The LTT measures the proliferation of T cells *in vitro* to *Borrelia* stimulation. Culture plates are precoated with recombinant *Borrelia* antigens [2]. Lymphocytes are then isolated from a whole blood sample and are incubated with the antigens for 5 days. Negatives controls and a positive control are included on each plate. After incubation, the cells are pulsed for 5 hours with 3 μC methyl-^{3}H-thymidine and the radioactivity is measured. Counts per minute are converted to a stimulation index (SI) representing the counts per minute in the tested well divided by the average counts per minute in negative control wells [2]. Results are interpreted following the manufacturer’s instruction. A cutoff of SI $\geq$ 3 in at least 1 well is defined as an equivocal result; two or more morphologically confirmed wells with SI $\geq$ 3 are considered a positive result.

2. Questionnaires

Questionnaires in this study are identical to the questionnaires used in the LymeProspect study [3]. They are described in that study’s protocol, as follows:

Fatigue severity is assessed by the fatigue severity subscale of the Checklist Individual Strength (CIS). [4] Scores range from 8 to 56, and scores of 35 or higher reflect severe fatigue. The Medical Outcomes Survey Short Form-36 (SF-36) pain subscale is used to assess severity and impact of pain. Significant impairment due to pain is reflected by a score of 55 or lower, based on Dutch norm scores. [5] Neurocognitive functioning is assessed with the Dutch version of the Cognitive Failure Questionnaire (CFQ). [6] Clinically significant complaints on neurocognitive functioning are reflected by a score of 44 or higher. Clinical and cognitive-behavioral parameters are assessed by online questionnaires as well, including somatic symptoms (PHQ-15) [7], physical and social functioning (SF-36, subscales physical functioning and social functioning) [8], health care use, absenteeism from work, co-morbidity (adapted from TiC-P) [9], pre-existent symptoms in the year preceding enrolment (adapted from PREDIS) [10], illness perception (adapted Brief IPQ) [11], cognitive and behavioral responses to symptoms (CBSQ) [12], psychological distress (HADS) [13], self-efficacy with respect to pain and fatigue (PCS, SEF-F) [14, 15], and the level of physical activity (IPAQ) [16].

3. Calculations of group sizes

Sensitivity and comparing the various cellular tests (t=0 weeks, LB cases):

Serological tests have a sensitivity of about 50% for early localized LB [17-19]. We consider cellular tests to have added diagnostic value if their sensitivity would rise to 70% or higher. An exact binomial test with a nominal 0.050 two-sided significance level will have 97% power to detect the difference between the Null hypothesis proportion, π0 of 0.500 (serology a t=0) and the Alternative proportion, πA, of 0.700 (cellular tests at t=0) when the sample size is 100. If the improvement in sensitivity is only 10-15%, inclusion of 150 cases will suffice to assess the primary outcome, since an exact binomial test with a nominal 0.050 two-sided significance level will have 85% power to detect the difference between the Null hypothesis proportion, π0 of 0.500 (serology at t=0) and the Alternative proportion, πA, of 0.625 (cellular test at t=0) when the sample size is 150.

Test-of-cure (t=12 weeks, LB cases):

To assess the discriminating power of the cellular tests, we will evaluate the proportion of patients that are negative in the cellular tests at t=12 weeks compared to the serological tests. From an earlier study [1], we assume that roughly 25% of patients or fewer are positive in the cellular tests at t=12 weeks. In contrast, we estimate that at least 40% of patients have a positive serological test. An exact binomial test with a nominal 0.050 two-sided significance level will have 81% power to detect the difference between the Null hypothesis proportion, π0 of 0.4 (serology at t=12 weeks) and the Alternative proportion, πA, of 0.250 (cellular tests at t=12 weeks) when the sample size is 80. Even though the time point at t=12 weeks is optional for LB cases, based on previous experience, we expect participation to be sufficient to achieve this goal.

Specificity (healthy controls):

Serological tests have a specificity of approximately 95% for early LB in case control studies [19, 20]. We consider cellular tests to have added value if the specificity would not drop below 90%. An exact binomial test with a nominal 0.050 two-sided significance level will have 81% power to detect the difference between the Null hypothesis proportion, π0 of 0.950 (serology) and the Alternative proportion, πA, of 0.90 (cellular tests) when the sample size is 225.

Other groups

No sample size calculation was made for the potentially cross-reactive controls and the observational cohort of patients with persistent signs and symptoms attributed to LB, because these analyses are descriptive. We aim to include a total of 60 potentially cross-reactive controls and 150 participants in the observational cohort.

4. References

1. Callister SM, Jobe DA, Stuparic-Stancic A, Miyamasu M, Boyle J, Dattwyler RJ, Arnaboldi PM: **Detection of IFN-gamma Secretion by T Cells Collected Before and After Successful Treatment of Early Lyme Disease**. *Clin Infect Dis* 2016, **62**(10):1235-1241.
2. Valentine-Thon E, Ilsemann K, Sandkamp M: **A novel lymphocyte transformation test (LTT-MELISA) for Lyme borreliosis**. *Diagn Microbiol Infect Dis* 2007, **57**(1):27-34.
3. Vrijmoeth HD, Ursinus J, Harms MG, Zomer TP, Gauw SA, Tulen AD, Kremer K, Sprong H, Knoop H, Vermeeren YM *et al*: **Prevalence and determinants of persistent symptoms after treatment for Lyme borreliosis: study protocol for an observational, prospective cohort study (LymeProspect)**. *Bmc Infectious Diseases* 2019, **19**.
4. Worm-Smeitink M, Gielissen M, Bloot L, van Laarhoven HWM, van Engelen BGM, van Riel P, Bleijenberg G, Nikolaus S, Knoop H: **The assessment of fatigue: Psychometric qualities and norms for the Checklist individual strength**. *J Psychosom Res* 2017, **98**:40-46.
5. Aaronson N, Muller M, Cohen P, Essink-Bot M, Fekkes M, Sanderman R, Sprangers M, te Velde A, E V: **Translation, validation, and norming of the Dutch language version of the SF-36 Health Survey in community and chronic disease populations.** *J Clin Epidemiol* 1998, **51**(11):1055-1068.
6. Ponds R, Van Boxtel M, Jolles J: **De Cognitive Failure Questionnaire als maat voor subjectief cognitief functioneren.** *Tijdschrift voor Neuropsychologie* 2006, **2**:37-45.
7. Stegenga BT, Kamphuis MH, King M, Nazareth I, Geerlings MI: **The natural course and outcome of major depressive disorder in primary care: the PREDICT-NL study**. *Soc Psychiatry Psychiatr Epidemiol* 2012, **47**(1):87-95.
8. Stewart JW, Quitkin FM, McGrath PJ, Rabkin JG, Markowitz JS, Tricamo E, Klein DF: **Social functioning in chronic depression: effect of 6 weeks of antidepressant treatment**. *Psychiatry Res* 1988, **25**(2):213-222.
9. Bouwmans C, De Jong K, Timman R, Zijlstra-Vlasveld M, Van der Feltz-Cornelis C, Tan Swan S, Hakkaart-van Roijen L: **Feasibility, reliability and validity of a questionnaire on healthcare consumption and productivity loss in patients with a psychiatric disorder (TiC-P)**. *BMC Health Serv Res* 2013, **13**:217.
10. Heins MJ, Knoop H, Lobbestael J, Bleijenberg G: **Childhood maltreatment and the response to cognitive behavior therapy for chronic fatigue syndrome**. *J Psychosom Res* 2011, **71**(6):404-410.
11. Broadbent E, Petrie KJ, Main J, Weinman J: **The brief illness perception questionnaire**. *J Psychosom Res* 2006, **60**(6):631-637.
12. Skerrett TN, Moss-Morris R: **Fatigue and social impairment in multiple sclerosis: the role of patients' cognitive and behavioral responses to their symptoms**. *J Psychosom Res* 2006, **61**(5):587-593.
13. Zigmond AS, Snaith RP: **The hospital anxiety and depression scale**. *Acta Psychiatr Scand* 1983, **67**(6):361-370.
14. Sullivan MJL, Bishop SR, Pivik J: **The Pain Catastrophizing Scale: Development and validation**. *Psychological Assessment* 1995, **7**(4):524-532.
15. Nicholas MK: **Self-Efficacy and Chronic Pain**. *Annual Conference of the British Psychological Society, St Andrews* 1989.
16. Craig CL, Marshall AL, Sjostrom M, Bauman AE, Booth ML, Ainsworth BE, Pratt M, Ekelund U, Yngve A, Sallis JF *et al*: **International physical activity questionnaire: 12-country reliability and validity**. *Med Sci Sports Exerc* 2003, **35**(8):1381-1395.
17. Nadelman R, Nowakowski J, Forseter G, al e: **The clinical spectrum of early lyme borreliosis in patients with culture-confirmed erythema migrans**. *The American Journal of Medicine* 1996, **100**(5):502-508.

18. Hansen K, Asbrink E: **Serodiagnosis of erythema migrans and acrodermatitis chronica atrophicans by the Borrelia burgdorferi flagellum enzyme-linked immunosorbent assay.** *Journal of Clinical Microbiology* 1989, **27**(3):545-551.
19. Leeflang MM, Ang CW, Berkhout J, Bijlmer HA, Van Bortel W, Brandenburg AH, Van Burgel ND, Van Dam AP, Dessau RB, Fingerle V *et al*: **The diagnostic accuracy of serological tests for Lyme borreliosis in Europe: a systematic review and meta-analysis**. *BMC infectious diseases* 2016, **16**:140.
20. Healthcare CIfQo: **Dutch guideline for Lyme disease**. *Available on https://wwwrivmnl/sites/default/files/2018-11/CBO%20richtlijn%20Lymeziekte%20definitief%20juli%202013pdf* 2013.
